# Delirium is associated with frequency band specific dysconnectivity in intrinsic connectivity networks: preliminary evidence from a large retrospective pilot case-control study

**DOI:** 10.1186/s40814-018-0388-z

**Published:** 2019-01-07

**Authors:** Robert Fleischmann, Steffi Traenkner, Antje Kraft, Sein Schmidt, Stephan J. Schreiber, Stephan A. Brandt

**Affiliations:** 1 Vision and Motor System Research Group, Department of Neurology, Charité – Universitätsmedizin Berlin, 10117 Berlin, Germany; 2 Department of Neurology, University Medicine Greifswald, 17475 Greifswald, Germany; 3 Department of Neurology, Asklepios Fachklinikum Brandenburg, 14772 Brandenburg an der Havel, Brandenburg Germany

**Keywords:** Delirium, Oscillatory activity, electroencephalography, Source analysis, Biomarker

## Abstract

**Background:**

Pathophysiological concepts in delirium are not sufficient to define objective biomarkers suited to improve clinical approaches. Advances in neuroimaging have revalued electroencephalography (EEG) as a tool to assess oscillatory network activity in neuropsychiatric disease. Yet, research in the field is limited to small populations and largely confined to postoperative delirium, which impedes generalizability of findings and planning of prospective studies in other populations. This study aimed to assess effect sizes of connectivity measures in a large mixed population to demonstrate that there are measurable EEG differences between delirium and control patients.

**Methods:**

This retrospective pilot study investigated EEG measures as biomarkers in delirium using a case-control design including patients diagnosed with delirium (DSM-5 criteria) and age-/gender-matched controls drawn from a database of 9980 patients (*n* = 129 and 414, respectively). Assessors were not blinded for groups. Power spectra and connectivity estimates, using the weighted phase log index, of continuous EEG data were compared between conditions. Alterations of information flow through nodes of intrinsic connectivity networks (ICN; default mode, salience, and executive control network) were evaluated in source space using betweenness centrality. This was done frequency specific and network nodes were defined by the multimodal human cerebral cortex parcellation based on human connectome project data.

**Results:**

Delirium and control patients exhibited distinct EEG power, connectivity, and network characteristics (*F*_(72,540)_ = 70.3, *p* < .001; *F*_(493,1079)_ = 2.69, *p* < .001; and *F*_(718,2159)_ = 1.14, *p* = .007, respectively). Connectivity analyses revealed global alpha and regional beta band disconnectivity that was accompanied by theta band hyperconnectivity in delirious patients. Source and network analyses yielded that these changes are not specific to single intrinsic connectivity networks but affect multiple nodes of networks engaged in level of consciousness, attention, working memory, executive control, and salience detection. Effect sizes were medium to strong in this mixed population of delirious patients.

**Conclusions:**

We quantified effect sizes for EEG connectivity and network analyses to be expected in delirium. This study implicates that theta band hyperconnectivity and alpha band disconnectivity may be essential mechanisms in the pathophysiology of delirium. Upcoming prospective studies will build upon these results and evaluate the clinical utility of identified EEG measures as therapeutic and prognostic biomarkers.

**Electronic supplementary material:**

The online version of this article (10.1186/s40814-018-0388-z) contains supplementary material, which is available to authorized users.

## Background

Delirium describes an acute confusional state that affects about 10–70% of hospitalized patients with the risk increasing with patient age and disease severity [1, 2]. Although it is generally considered a reversible condition, diagnosing and treating affected patients to avoid short- and long-term complications remains a challenge, particularly given misdiagnosis rates of up to 76% [3, 4]. This situation is not least due to an insufficient understanding of underlying pathophysiological mechanisms precluding other than symptom-oriented interventions [5–7]. In line with this notion, therapeutic approaches in delirium are largely unstandardized albeit adequate treatment can prevent or mitigate unfavorable outcomes including persistent cognitive impairment [8–11]. A robust pathophysiological concept would help establish biomarkers, i.e. objective measures of the condition, which can facilitate the diagnostic process and guide standardized and targeted approaches [12].

Current research indicates that electroencephalography (EEG) is a promising tool and suitable means to elaborate on the neurophysiological basis of delirium [13, 14]. Several studies have already underlined its utility to provide diagnostic biomarkers, yet few studies harnessed advanced EEG analyses to elaborate on its neurophysiological foundations [15, 16]. Important benefits of EEG in this context are its ready availability, safety, ease to use, and high temporal resolution including the possibility to correct for artifacts during and after acquisition. Recent advances in temporal, spatial, and connectivity analyses have furthermore significantly enhanced and revalued its potential to image brain function and study unobservable brain states, i.e. biomarkers of cognitive function [17]. This includes the investigation of intrinsic connectivity networks (ICN), which can be studied in a task-free resting or free-driving cognitive state and hence enable the examination of brain activity in a number of neuropsychiatric diseases that are not amenable to complex cognitive subtraction designs [18, 19]. The paucity of neuroimaging, particularly electrophysiogical, studies in delirium is even more surprising since oscillatory network activity has long been recognized to be not only a by-product of neuronal activity, but to serve communication in the brain that can lead to a wide spectrum of behavioral disorders when its homeostasis is disturbed [20, 21]. Few exceptional studies investigated measures of functional and directional connectivity using EEG or functional magnetic resonance imaging (fMRI) in delirium. These studies reported changes in the default mode network, salience network, and executive control network to underly disturbances of cognition and consciousness in the acute state and delayed cognitive impairment [22–28]. EEG studies furthermore revealed increased spectral variability, decreased complexity, loss of alpha band connectivity, increased delta band connectivity including enhanced information flow from posterior to anterior regions, and decreased path length [23, 29, 30]. Results yet need to be interpreted with caution given several limitations. Patient cohorts were generally small, including 20 patients or less, and either recruited patients with postoperative delirium following cardiothoracic surgery or did not state the cause of delirium [16, 31, 32]. In this context, it is important to note that brain regions were shown to be differently affected by delirium of varied causes and in different populations, which limits the generalizability of results from small cohorts [33–35]. Particular motor subtypes could furthermore be overrepresented, which is known to significantly influence connectivity signatures [30, 32]. Investigations of restricted patient populations may therefore underly contradictory connectivity results of both increased and decreased functional connectivity with changes of directionality from posterior to anterior regions and vice versa [23, 24, 30].

Above-mentioned limitations render further investigations of EEG measures as biomarkers of cognitive dysfunction in delirium challenging. Importantly, effect sizes to be expected in mixed or other than cardiothoracic populations cannot be deduced. It is the objective of this study to estimate effect sizes of EEG connectivity measures in a mixed delirious population representative of patients treated in a large tertiary care hospital. This is a critical and mandatory step to estimate sample sizes and resources required for a planned prospective study on the utility of advanced EEG measures as therapeutic and prognostic biomarkers of delirium. For this purpose, we evaluated resting state EEG data obtained during the routine clinical work-up of delirious patients that were confirmed by validated clinical tools. Results were compared to age- and gender-matched controls. The retrospective study design allowed us to include a large sample size that accounts for potentially high variance inherent to the heterogeneity of delirium subtypes. Connectivity analyses included the exploratory investigation of oscillatory activity changes in established ICNs, which are discussed regarding their implications for the pathophysiology of delirium.

## Materials and methods

### Study design and regulations

This is a pilot case-control study investigating effect sizes of EEG connectivity measures that differentiate between delirium and controls, which is a prerequisite for their subsequent evaluation as biomarkers in a prospective study [36, 37]. The study conformed to the *Helsinki declaration*. Data protection and ethics review committee approval were obtained from the Institutional Review Board of the Charité Universitätsmedizin Berlin in line with regulations for retrospective studies. This study furthermore conforms with the STROBE Statement reporting standard for case-control studies [38]. Aspects specific to the pilot character of the study are in line with the CONSORT extension for randomized pilot and feasibility trials, not considering items that are required for randomized but not case-control studies [39].

### Participants and selection of EEG recordings

Samples of delirious patients and control subjects with normal EEGs were drawn from the digital EEG database that included all EEGs acquired between the years 2004 and 2016 by the Department of Neurology at the Charité–Universitätsmedizin Berlin, Germany. Cases of delirium were identified in a stepwise approach including chart reviews based on DSM-5 criteria and the documented use of screening tools, which is expected to yield a high sensitivity and specificity for the detection of delirium even in a retrospective evaluation [40, 41]. All screenings of reports and charts were jointly conducted by two of the researchers (RF, ST), who unanimously decided whether a patient was classified delirious or not. A first screening was performed by searching EEG reports for keywords including “delir*,” “conscious*,” and “confusion*” (asterisks indicate wildcard characters). A detailed list of search terms is included in Additional file 1. Patients were excluded if reports indicated that interpretation was limited by artifacts, medication, or vigilance. Discharge letters of remaining patients were carefully reviewed, and subjects excluded if delirium was not clearly diagnosed with a validated screening tool and in accordance with DSM-5 criteria [42] or imaging indicated structural brain lesions (stroke, tumor, inflammatory disease, etc.). Age- and gender-matched controls were drawn from a population of patients with normal EEGs through an automated systematic sampling approach using MATLAB® (MATLAB 2008b, The Mathworks, Natick, MA, USA). Frequency matching was chosen over 1:1 matching because it was unclear how examined EEG parameters would be distributed in the control population and a larger control group would minimize the risk of randomly selecting a sample that was by chance confounded by another unknown condition. The main sampling criterion was that the probability of control samples to have the same mean age and variance as delirious patients (null hypothesis) is ≥ 99% in a *z* test and equal gender distribution within 10-year age intervals. A flow diagram of the complete patient selection procedure can be found in Additional file 2.

### EEG acquisition, processing, and spectral analysis

EEG were digitally recorded through a commercially available clinical EEG system with a sampling rate of 256 Hz (Galileo.NET, BE Light system, EB Neuro S.p.A., Firenze, Italy). Each recording lasted at least 20 min in line with International Federation of Clinical Neurophysiology recommendations for clinical EEGs [43]. Electrodes were positioned according to the 10–20 system. Preprocessing and analysis of EEG data were performed using FieldTrip, an open source software package that is implemented as MATLAB® toolbox and enables a broad spectrum of simple and advanced EEG analyses including source reconstruction and network analyses [44]. Standardized epochs of provocation (hyperventilation, photo stimulation) and eyes-open/eyes-closed maneuvers were excluded. Auricular electrodes were removed from the analysis. All channels were referenced to common average. Data preprocessing included detrending and application of discrete Fourier transform filtering at 50 Hz and its harmonics. Data was segmented into artifact-free trials of 10,000 ms. Trials containing artifacts were rejected semiautomatically. Trials containing excessive variance defined by a *z*-value threshold of 20 were removed. Remaining trials were then visually inspected for artifacts by an experienced EEG reader and rejected as necessary. EEG data was subsequently transformed to frequency space including frequencies of interest between 1 and 70 Hz using a multitaper method fast Fourier transform based on discrete prolate spheroidal sequences windowing. Grand averages were calculated for all trials of one subject. Subject-specific power spectra were standardized by their mean power to allow for comparison of frequency- and sensor-specific power distributions between subjects and groups.

### Connectivity and network analyses

Connectivity was analyzed on a sensor level to enable comparisons with previous EEG studies performed in delirium [23, 30]. Given that volume conduction is a major concern when performing analyses on a sensor level, previous studies used the phase lag index (PLI) as a measure that is, compared to other measures, relatively insensitive to volume conduction, common sources, and active reference electrodes [23, 45]. We used the weighted PLI (wPLI) that weighs the contribution of the observed phase leads and lags by the magnitude of the imaginary component of the cross-spectrum, which provides the advantages of reduced sensitivity to uncorrelated noise sources and increased statistical power to detect changes in phase-synchronization [46].

Source reconstruction was performed as a prerequisite for network analyses. Importantly, recent research confirmed the validity of source analyses based on electrode locations of the 10–20 system [18]. The underlying volume conductor model consisted of a boundary element model that was calculated from a T1-weighted template MRI with 1 mm resolution, which is an established and viable approach for source reconstructions in larger populations in which individual high resolution MRI are not available [47–49]. Whole-brain source reconstruction was performed using partial canonical coherence algorithms provided with the Fieldtrip toolbox [44]. Source connectivity was first calculated through coherence analyses based on the absolute imaginary part of the coherence spectrum, which effectively suppresses spurious coherence driven by volume conduction [50]. Network analyses of source data were then performed using betweenness centrality as a well-established graph theoretical measure that reflects the number of links incident upon a node and therefore reflects its importance [51]. The resulting functional map was subsequently parcellated into parcels defined by the multimodal human cerebral cortex parcellation based on human connectome project data [52]. For each parcel, we finally calculated the largest eigenvector of centrality parameters, which represents the main component of effects. Masks of well-established intrinsic connectivity networks, which were already studied in the context of delirium or expected to contribute to its pathophysiology (i.e., default mode, salience, and executive control networks), were finally applied to parcellated network data in order to compare results to previous studies including fMRI data and to facilitate their functional interpretation [19, 20, 24, 28, 53]. Regions of interest of networks used in this study are publicly available [54].

### Sample size considerations

Given the focus on hypothesis tests for the detection of potential EEG biomarkers in delirium, it is critical to show that sample sizes satisfy the identification of biomarkers with sufficient statistical power. Above-mentioned selection criteria yielded 129 patients with delirium and 414 control patients (more detailed patient characteristics are presented in “Patient characteristics” Section). G*power 3.1.9.2 was used to compute the resulting sensitivity, i.e. minimum effect sizes that can be identified (Heinrich-Heine-University, Düsseldorf, Germany). Two-tailed *t*-tests for the difference between two independent means would identify effect sizes *d* of .28 given an alpha error probability of 5% and power of 80%, which renders the identification of medium effect sizes possible.

### Statistics

Univariate three-way analysis of variance (ANOVA) was performed as a global test for significant main effects and interactions of power spectra, connectivity, and network data including the factors frequency band (FREQUENCY), delirious or control group (GROUP), and location of the EEG signal at different electrode sites (SENSOR) or within different regions, when source data were used (REGION). Frequency bands were grouped in delta (1–3 Hz), theta (4–7 Hz), alpha (8–13 Hz), beta (14–30 Hz), and gamma (31–70 Hz) activity. Significant ANOVA test results were post-hoc compared by Bonferroni-corrected marginal means. Connectivity and network analyses were performed by whole-brain Bonferroni-corrected independent two-tailed *t*-tests between the two conditions, i.e. delirium and control. Differences in connectivity were analyzed based on wPLI data for each sensor and frequency. Differences in network information flow were analyzed based on the largest eigenvector of betweenness centrality per parcel and frequency band. Effect sizes (Cohen’s *d*) were directly calculated from *t*-values and respective degrees of freedom. Effect sizes lower than .2 were considered small, between .2 and .5 medium, between .5 and .8 large, and those greater than .8 very large [55]. Effect sizes of potential biomarkers should be at least medium to strong to justify their investigations in prospective studies. Results of descriptive statistics are reported as differences of group means and their 95% confidence intervals (95% CI) in square brackets for simple spectral analyses. Assessors were not blinded for outcomes. Absolute values of descriptive statistics for connectivity and network analyses can be found in figures but are not written in text for the purpose of legibility. Results from inferential *t*-statistics are given as *t*-values, *p*-values, and their respective effect size. *p*-values lower than .05 were considered significant; those lower than .001 are not reported exact but as < .001.

## Results

### Patient characteristics

EEG reports and data of 9980 patients were screened. We included EEG data from 129 patients that met the inclusion criteria for the delirium group (mean age 73.6 years ± 13.9; 43% female). The control group consisted of EEG data from 414 age- and gender-matched patients with unremarkable EEG reports (mean age 73.6 years ± 13.9; 43% female). Both groups did not differ with respect to age (*p* = .99) or male to female ratio (*p* = .97). Causes of delirium were sepsis (*n* = 26), surgery (*n* = 24), metabolic disturbances (*n* = 18), central nervous infections (*n* = 15), sepsis following surgery (*n* = 10), single other causes (*n* = 17), or unclear (*n* = 19). Indications for an EEG examination in control cases were evaluations of epileptic activity (*n* = 99), syncopations (*n* = 53), and cognitive decline (*n* = 47) among others. All patients were treated as inpatients and in -hospital allocations were (number of delirium/control patients): neurology ward (64/248), neurological intensive care unit (50/112), intermediate care unit (8/42), and emergency department (7/12).

### Sensor level results

ANOVA of power spectra revealed a significant main effect for GROUP (*F*_(1,542)_ = 714.5, *p* < .001), SENSOR (*F*_(18,542)_ = 118.3, *p* < .001), and FREQUENCY (*F*_(4,542)_ = 4039.9, *p* < .001), i.e. power spectra were significantly different between delirious and control patients and also differed between frequencies. Differences were not limited to main effects but power spectra also differed between delirious and control patients for individual frequencies (GROUP × FREQUENCY (*F*_(4,541)_ = 840.5, *p* < .001)), at individual EEG electrode locations (GROUP × SENSOR (*F*_(18,541)_ = 174.5, *p* < .001)), and individual frequencies at individual EEG electrode locations (GROUP × SENSOR × FREQUENCY (*F*_(72,540)_ = 70.3, *p* < .001)). Post-hoc analyses yielded an increase in total delta (8.89 μV^2^/Hz [8.61–9.17], *p* < .001) and theta (1.93 μV^2^/Hz [1.65–2.21], *p* < .001) power in the delirium group. Alpha (− 1.66 μV^2^/Hz [− 1.94–− 1.38], *p* < .001) and beta (− 0.42 μV^2^/Hz [− 0.70–− 0.14], *p* = .004) power were significantly decreased while gamma power was unchanged between conditions (*p* = .604). Frequency-specific effects at distinct electrode sites between conditions were further elaborated on by pairwise comparisons. Most prominent findings were an increase in frontal delta and theta power, decreased occipital alpha power, and decreased parieto-occipital beta power accentuated in the left hemisphere in the delirium group. A full table of results can be found in Additional file 3.

**Fig. 1:**
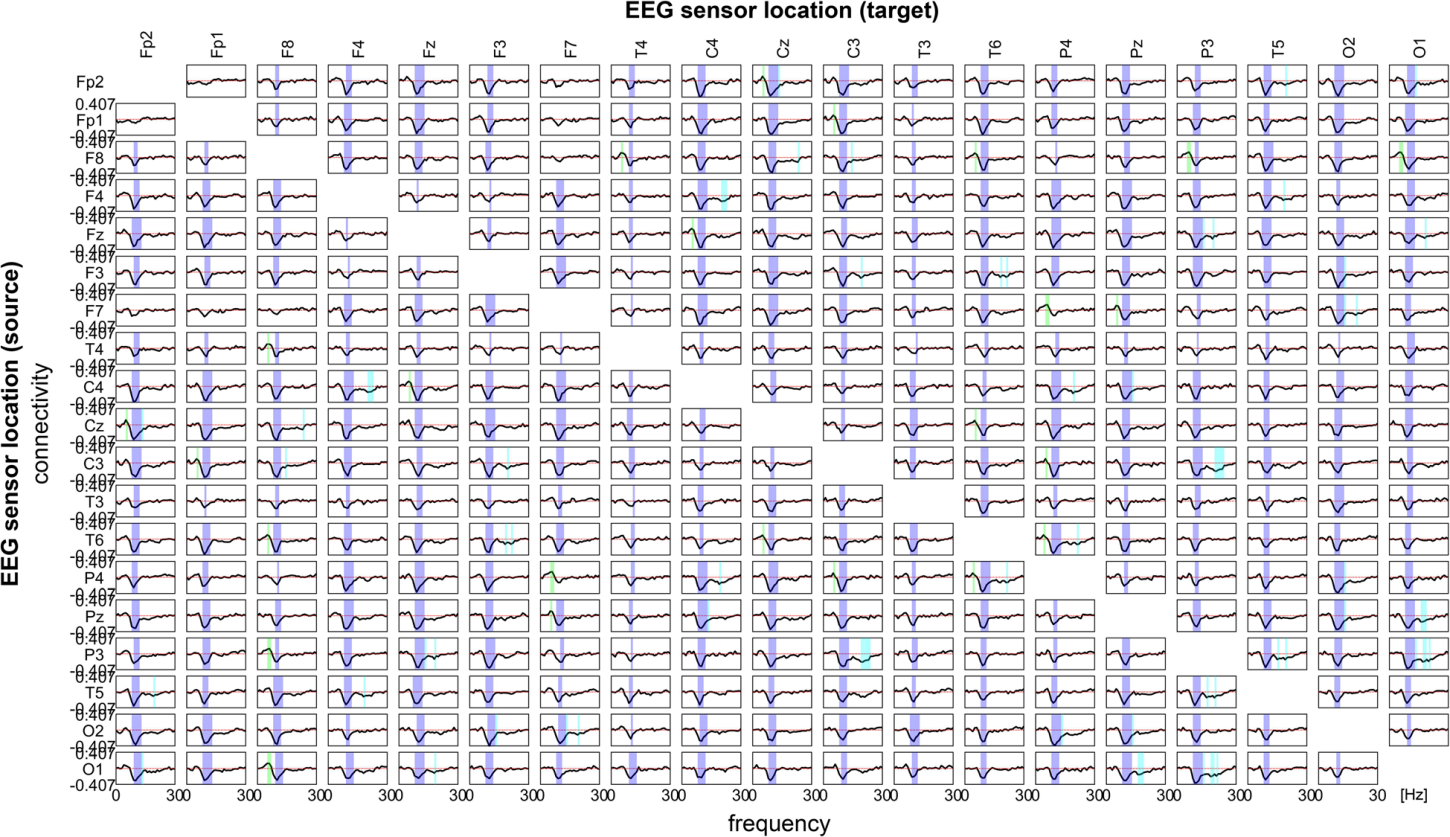
Difference of sensor level connectivity between groups. Sensors of the 10–20 EEG system used in this study are shown in columns and rows. Their connectivity changes in delirium are illustrated with frequencies on the *x*-axis and weighted phase lag indices on the *y*-axis. Values greater than 0 on the *y*-axis indicate increased connectivity in delirium while lower values indicate decreased connectivity. Changes in the delta band were not significant after Bonferroni correction for alpha error accumulation. Significant changes in the theta, alpha, and beta band are indicated by green, purple, and blue bars in the background, respectively. The red dotted line represents zero change. Note that changes in the theta and beta band are more localized while alpha band connectivity is globally changed

ANOVA also revealed a significant interaction of connectivity estimates for GROUP × SENSOR × FREQUENCY (*F*_(493,1079)_ = 2.69, *p* < .001), i.e. connectivity significantly differed between delirious and control patients, and also differed between frequencies and EEG electrode locations. Results are summarized in Fig. 1. Connectivity in the delta band remained largely unchanged, while there were significant regional changes in the theta and beta band. Alpha band connectivity was globally reduced. Regional increases in theta band connectivity were particularly apparent in the right central parieto-temporal region (P4–T6, *t*_(541)_ = 4.95, *p* = .04; Cohen’s *d* = .43 [.39–.47]; P4–F7, *t*_(541)_ = 4.71, *p* = .04, Cohen’s *d* = .4 [.37–.43]; C4–Cz, *t*_(541)_ = 4.36, *p* = .05, Cohen’s *d* = .37 [.33–.41]). Decreased connectivity in the beta band was most prominent in parieto-occipital regions (Pz–O1, *t*_(541)_ = − 6.22, *p* < .001, Cohen’s *d* = − .53 [− .57–− .49]; P3–O1, *t*_(541)_ = − 4.65, *p* = .03, Cohen’s *d* = − .4 [− .45–− .35]; P3–C3, *t*_(541)_ = − 4.57, *p* = .03, Cohen’s *d* = − .39 [− .44–− .34]). Strongest reductions in alpha connectivity were found between Fp2–C4 and Fp2–Cz (*t*_(541)_ = − 11.57, *p* < .001, Cohen’s *d* = − 1 [− .1.06–− .94] and *t*_(541)_ = − 10.24, *p* < .001,, Cohen’s *d* = − .88 [− .94–− .82], respectively).

### Network analysis

**Fig. 2:**
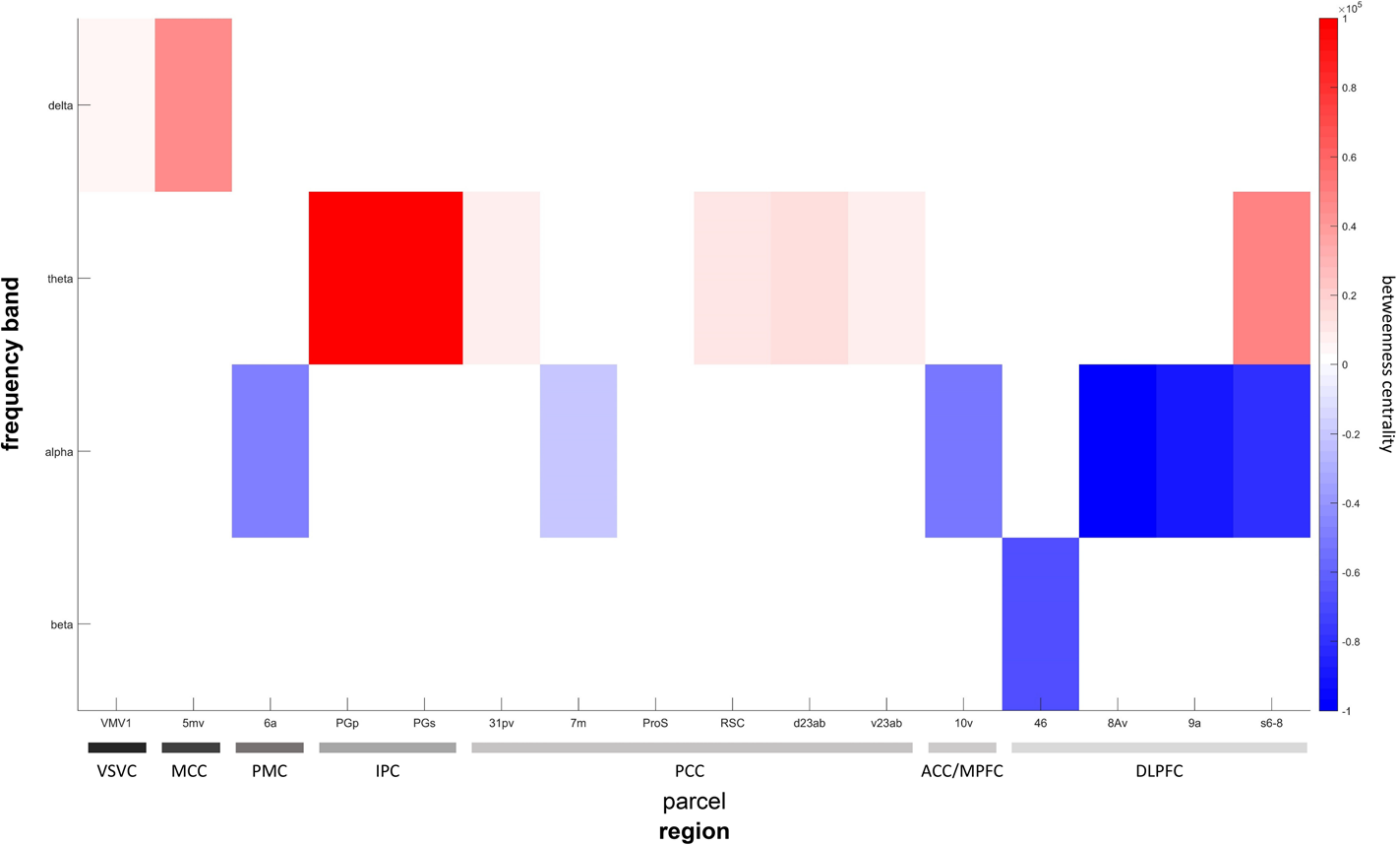
Betweenness centrality differences between groups in the default mode network. Parcels located in the default mode network and representing the ninth percentile of changes in betweenness centrality between groups based on their *t*-values are shown. Rows represent frequency bands, columns represent parcels. Parcels are grouped by regions as indicated by gray scaled horizontal bars based on their definition by Glasser et al. [52]. Red and blue colors represent an increase or a decrease of betweenness centrality, respectively. Note that there is an increase throughout nodes in the delta and theta band while alpha and beta band centrality decreased. Abbreviations for regions: *VSVC* ventral stream visual cortex, *MCC* paracentral lobular and mid cingulate cortex, *PMC* premotor cortex, *IPC* inferior parietal cortex, *PCC* posterior cingulate cortex, *ACC*/*MPFC* anterior cingulate and medial prefrontal cortex, *DLPFC* dorsolateral prefrontal cortex

There was a significant interaction of GROUP × REGION × FREQUENCY for the ANOVA of betweenness centrality estimates (*F*_(718,2159)_ = 1.14, *p* = .007). This indicates that information flow between nodes of examined networks differs between delirious and control patients, and that this effect is further specified by oscillatory frequencies. A summary of findings is given in Table 1. In general, all networks showed increased centrality parameters in slow frequencies and decreased centrality in faster frequencies. Multiple nodes of the default mode network (DMN) showed changes of their betweenness centrality as illustrated in Fig. 2. Most prominent changes throughout most frequency bands were found in the dorsolateral prefrontal cortex (DLPFC) and posterior cingulate cortex (PCC). Delta and theta centrality changes were inversely related to changes of faster frequencies that exhibited decreased centrality. In the DLPFC, information flow in the theta band was significantly enhanced (parcel s6–8, *t*_(541)_ = 4.26, *p* < .001, Cohen’s *d* = .37 [.31–.41]) while alpha (e.g., parcel 8Av, *t*_(541)_ = − 3.73, *p* < .001, Cohen’s *d* = − .32 [− .36–− 26]) and beta (parcel 46, *t*_(541)_ = − 2.36, *p* = .01, Cohen’s *d* = − .2 [− .22–− .18]) band centrality were decreased. Similar changes were found in the PCC where delta band centrality was increased (parcel ProS, *t*_(541)_ = 2.99, *p* = .001, Cohen’s *d* = .26 [.23–.28]) in line with changes in the theta band (e.g., parcel RSC, *t*_(541)_ = 5.47, *p* < .001, Cohen’s *d* = .47 [.36–.55]) while faster frequencies revealed only decreased centrality in the alpha band (parcel 7m, *t*_(541)_ = − 3.95, *p* < .001, Cohen’s *d* = − .34 [− .39–− .27]).

**Table 1:** Summary of betweenness centrality analyses

Network	Frequency	HCP-MMP region	HCP-MMP location	*t*-value	*p*-value	Cohen’s *d*	Cohen’s *d* [95% CI]
Default mode network	Delta	paracentral lobular and mid cingulate cortex	5mv	2.44	0.01	0.21	0.19–0.22
		posterior cingulate cortex	ProS	3.00	0.00	0.26	0.23–0.28
		ventral stream visual cortex	VMV1	2.02	0.02	0.17	0.16–0.18
	Theta	posterior cingulate cortex	RSC	5.47	0.00	0.47	0.36–0.55
		posterior cingulate cortex	v23ab	5.41	0.00	0.47	0.36–0.54
		posterior cingulate cortex	d23ab	4.29	0.00	0.37	0.3–0.42
		posterior cingulate cortex	31 pv	5.58	0.00	0.48	0.37–0.55
		dorsolateral prefrontal cortex	s6–8	4.26	0.00	0.37	0.31–0.41
		inferior parietal cortex	PGp	4.18	0.00	0.36	0.31–0.4
		inferior parietal cortex	PGs	5.52	0.00	0.47	0.38–0.54
	Alpha	posterior cingulate cortex	7 m	− 3.95	0.00	− 0.34	− 0.39–− 0.27
		dorsolateral prefrontal cortex	8Av	− 3.73	0.00	− 0.32	− 0.36–− 0.26
		dorsolateral prefrontal cortex	9a	− 3.44	0.00	− 0.3	− 0.33–− 0.25
		anterior cingulate and medial prefrontal cortex	10v	− 3.44	0.00	− 0.3	− 0.33–− 0.25
		premotor cortex	6a	− 3.72	0.00	− 0.32	− 0.36–− 0.26
		dorsolateral prefrontal cortex	s6–8	− 3.32	0.00	− 0.29	− 0.32–− 0.24
	Beta	dorsolateral prefrontal cortex	46	− 2.36	0.01	− 0.2	− 0.22–− 0.18
Executive control network	Delta	superior parietal cortex	MIP	1.86	0.03	0.16	0.15–0.17
	Theta	dorsolateral prefrontal cortex	s6–8	4.26	0.00	0.37	0.31–0.41
		lateral temporal cortex	TE2a	4.77	0.00	0.41	0.34–0.46
		inferior parietal cortex	IP2	4.71	0.00	0.41	0.33–0.46
		inferior parietal cortex	PF	4.38	0.00	0.38	0.32–0.42
		inferior parietal cortex	PGs	5.52	0.00	0.47	0.38–0.54
	Alpha	dorsolateral prefrontal cortex	8Av	− 3.73	0.00	− 0.32	− 0.36–− 0.26
		dorsolateral prefrontal cortex	9a	− 3.44	0.00	− 0.30	− 0.33–− 0.25
		premotor cortex	6a	− 3.72	0.00	− 0.32	− 0.36–− 0.26
		dorsolateral prefrontal cortex	s6–8	− 3.32	0.00	− 0.29	− 0.32–− 0.24
	Beta	dorsolateral prefrontal cortex	46	− 2.36	0.01	− 0.20	− 0.22–− 0.18
Salience network	Delta	anterior cingulate and medial prefrontal cortex	p32pr	1.66	0.05	0.14	0.14–0.15
		anterior cingulate and medial prefrontal cortex	8BM	3.90	0.00	0.34	0.29–0.37
		dorsolateral prefrontal cortex	8Ad	3.74	0.00	0.32	0.28–0.35
	Theta	dorsolateral prefrontal cortex	s6–8	4.26	0.00	0.37	0.31–0.41
		anterior cingulate and medial prefrontal cortex	p24	3.70	0.00	0.32	0.27–0.36
		dorsolateral prefrontal cortex	9-46d	− 3.30	0.00	− 0.28	− 0.31–− 0.24
	Alpha	dorsolateral prefrontal cortex	9a	− 3.44	0.00	− 0.30	− 0.33–− 0.25
		dorsolateral prefrontal cortex	s6–8	− 3.32	0.00	− 0.29	− 0.32–− 0.24
	Beta	dorsolateral prefrontal cortex	46	− 2.36	0.01	− 0.20	− 0.22–− 0.18

All networks studied showed alterations in information flow through multiple nodes and throughout frequency bands. Slow frequencies were generally associated with increased centrality while faster frequencies rendered decreased centrality parameters. Note that only *t*-values above the 9th percentile were included to illustrate largest effects. Effect sizes were medium (Cohens *d* = 0.3–0.6) in most cases. For a full list of HCP-MMP locations and their associated functions, please refer to the original publication by Glasser et al. [52]

**Fig. 3:**
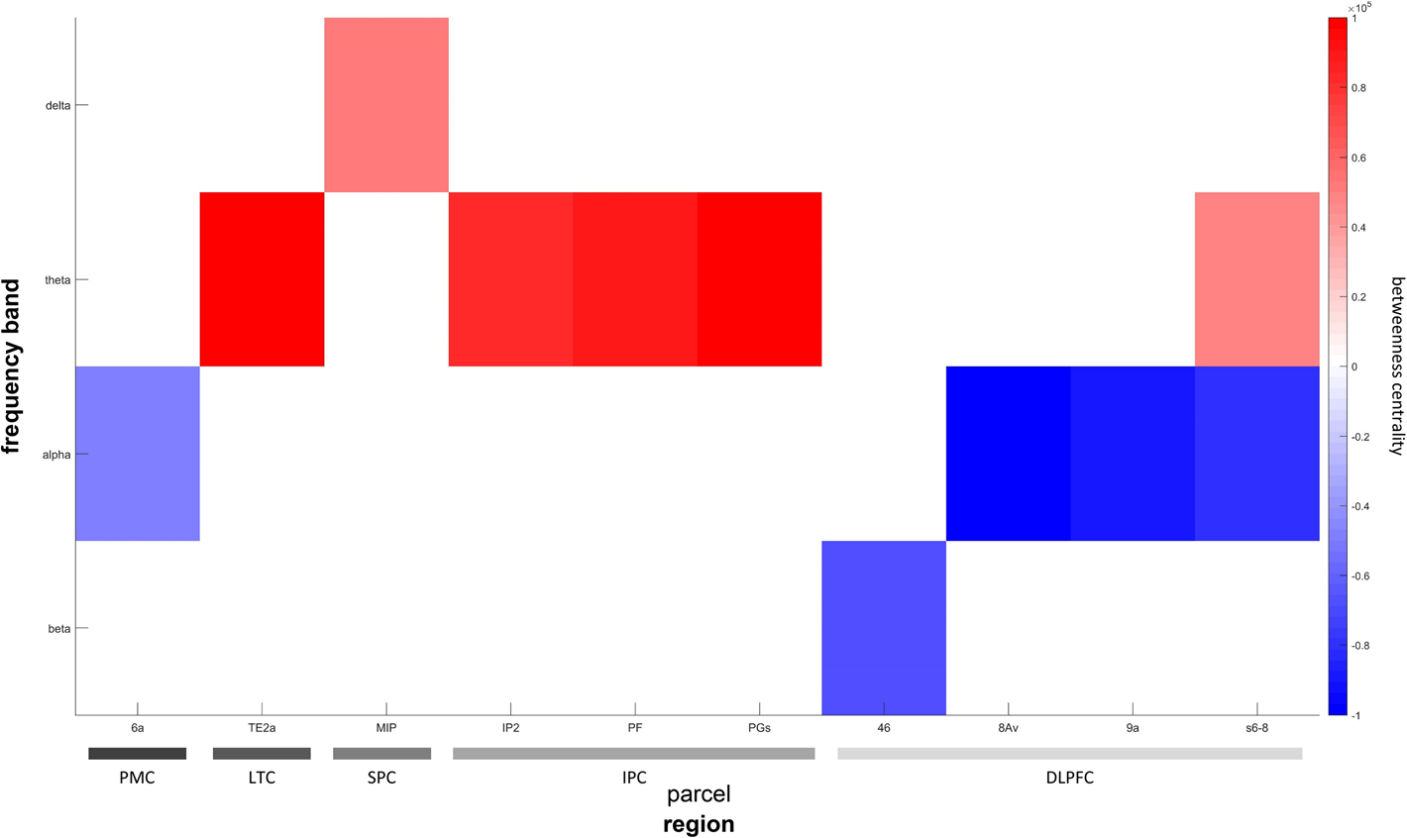
Betweenness centrality differences between groups in the executive control network. Parcels located in the executive control network and representing the ninth percentile of changes in betweenness centrality between groups based on their *t*-values are shown. Definition of rows and columns is analogue to the description of Fig. 2. Like in the default mode network, there is an increase throughout nodes in the delta and theta band while alpha and beta band centrality decreased. Abbreviations for regions: *PMC* premotor cortex, *LTC* lateral temporal cortex, *SPC* superior parietal cortex, *IPC* inferior parietal cortex, *DLPFC* dorsolateral prefrontal cortex

Centrality parameters of the executive control network (ECN) showed alterations throughout frequency bands that are summarized in Fig. 3. Betweenness centrality in the parietal cortex was significantly enhanced for slow frequencies while faster frequencies did not exhibit significant changes. The inferior parietal cortex revealed increased centrality in the theta band (e.g., parcel PGs, *t*_(541)_ = 5.52, *p* < .001, Cohen’s *d* = 47 [.38–.54]) while enhanced delta band information flow was found in parcel MIP of the superior parietal cortex (*t*_(541)_ = 1.86, *p* = .03, Cohen’s *d* = .16 [.15–.17]). The DLPFC, in line with changes in the DMN, showed increased centrality in the theta band (parcel s6–8, *t*_(541)_ = 4.23, *p* < .001, Cohen’s *d* = .37 [.31–.41]) while faster frequencies in the alpha (parcel 8Av, *t*_(541)_ = − 3.73, *p* < .001, Cohen’s *d* = −.32 [− .36–− .26]) and beta (parcel 46, *t*_(541)_ = − 2.36, *p* = .01, Cohen’s *d* = − .2 [− .22–− .18]) band revealed decreased centrality.

**Fig. 4:**
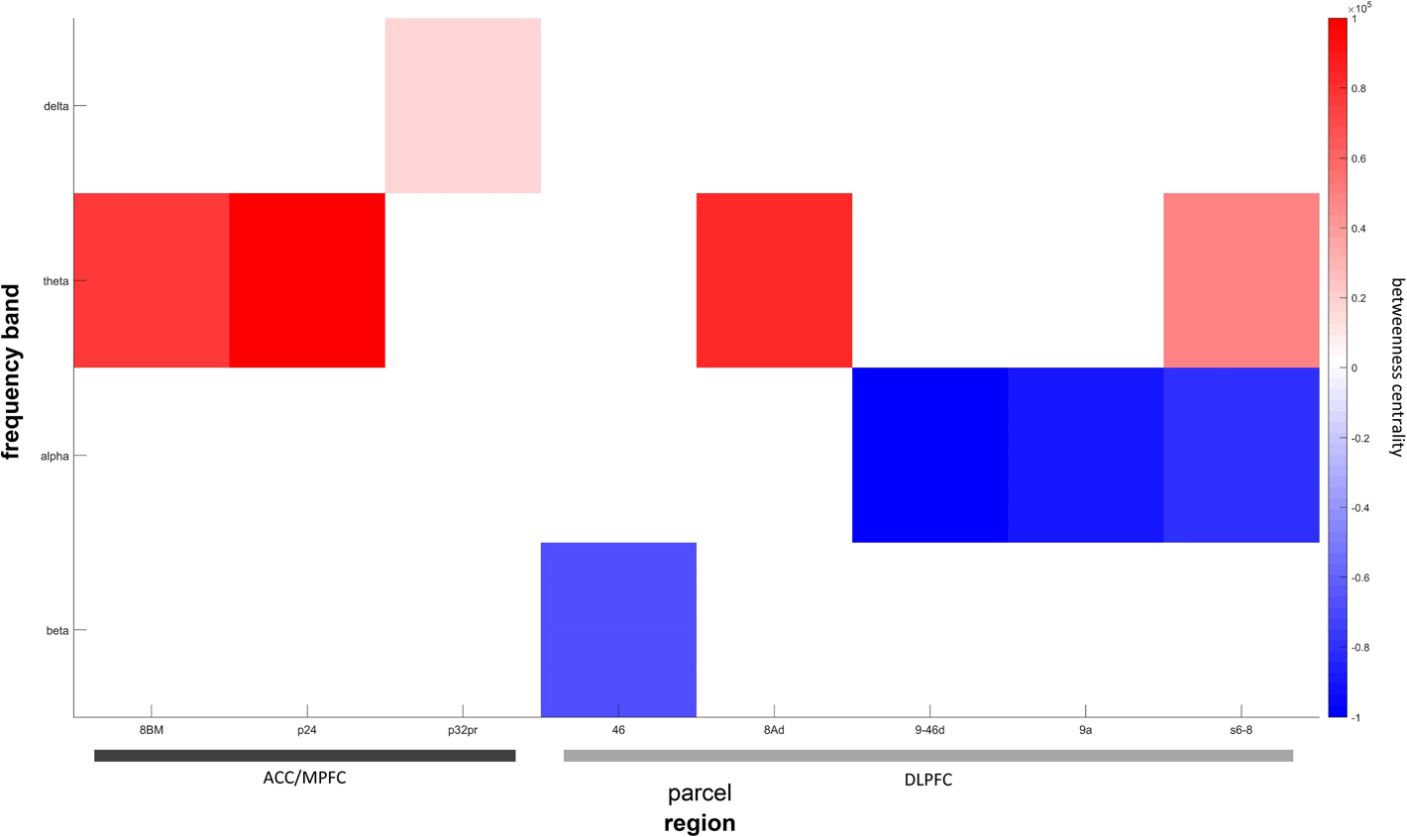
Betweenness centrality differences between groups in the salience network. Parcels located in the salience network and representing the ninth percentile of changes in betweenness centrality between groups based on their *t* values are shown. Definition of rows and columns is analogue to the description of Fig. 2. Alpha and beta band centrality is decreased throughout while there is a consistent increase in the delta and theta band. *ACC*/*MPFC* anterior cingulate and medial prefrontal cortex, *DLPFC* dorsolateral prefrontal cortex

Only two regions of the salience network revealed changes in centrality. The anterior cingulate cortex/medial prefrontal cortex (ACC/MPFC) was exclusively altered regarding slow frequencies. Delta band centrality was only increased in parcel p32pr (*t*_(541)_ = 1.66, *p* = .05, Cohen’s *d* = .14 [.14–.15]) while centrality in the theta band was enhanced in two parcels with maximum changes in parcel 24 (*t*_(541)_ = 3.7, *p* < .001, Cohen’s *d* = .32 [.28–.35]). Changes in the DLPFC were in line with changes in the DMN and ECN confined to the theta (parcel s6–8, *t*_(541)_ = 4.26, *p* < .001, Cohen’s *d* = .37 [.31–.41]), alpha (parcel 9a, *t*_(541)_ = − 3.44, *p* < .001, Cohen’s *d* = − .3 [− .33–− .25]), and beta band (parcel 46, *t*_(541)_ = − 2.36, *p* = .01, Cohen’s *d* = − .2 [− .22–− .18]) while delta band centrality was unchanged between conditions. A summary of findings is given in Fig. 4.

## Discussion

This is the first study to elaborate on altered oscillatory brain activity not only in a specific subgroup but in a large cohort of delirious patients of mixed etiologies and to integrate findings from connectivity and source analyses into an intrinsic connectivity network context. We found altered sensor level connectivity throughout frequency bands including beta band activity that was previously not reported in studies investigating delirium pathophysiology. Simple spectral analyses confirmed previous results of increased slow oscillatory activity and decreased alpha activity. Connectivity analyses revealed global disconnectivity in the alpha band that was paralleled by a hyperconnectivity within the theta band in delirious patients. Source and network analyses revealed that these changes are not specific to single intrinsic connectivity networks but affect multiple nodes of networks engaged in level of consciousness, attention, working memory, executive control, and salience detection. We were furthermore able to estimate effect sizes that were generally medium to strong in this mixed population of delirious patients, which supports the notion of EEG as an excellent method for biomarkers in delirium.

### Comparison of simple spectral analyses to previous EEG studies in delirium

Recent reviews on EEG changes in delirium include ambiguous reports of changes of all slow frequency oscillations, increased activity only in the lower or total theta band, or decreased activity in the alpha band [16]. Our results from a mixed cohort of patients support the view that delirium can be characterized by power increase throughout slow frequencies and decrease throughout faster oscillatory activity. This result may indicate that previous studies investigated neurophysiological subtypes of delirium that are defined by subsets of oscillatory activity changes in confined populations and that such delimitations fade in a mixed population. Another possibility is that differences reflect severity of delirious states or temporal evolution throughout the course of the disorder. In any case and given that a direct link between neurotransmission and delirium pathogenesis is proven, prospective studies will clarify the significance of global power changes as neurophysiological biomarkers [56, 57].

### Sensor level connectivity

Alterations in network connectivity have repeatedly been proposed to underly behavioral disturbances in delirium [25, 53, 58]. Given the wide acceptance of this hypothesis, it is surprising that few studies investigated connectivity changes in delirium. An exception to this notion is the study conducted by van Dellen et al. in patients following cardiac surgery [23]. They analyzed data from a 21-channel routine EEGs and found decreased posterior-anterior connectivity and lower alpha band network integrity compared to normal controls during hypoactive delirium. Delta band connectivity was increased toward frontal regions. Other frequency bands were unaffected. Similar findings were recently reported in a group of patients recovering from anesthesia [30]. Numan et al. used the directed phase transfer entropy to estimate the direction of information flow and confirmed a disturbed back-to-front connectivity in the alpha band that is thought to underly disturbances of consciousness [23, 30, 59]. While we did not find disturbed connectivity in the delta band, possibly due to the heterogeneity of the study population, we found substantial global disconnectivity in the alpha band that was most pronounced between central and frontal sensor locations. Effect sizes were stronger than in any other frequency band indicating the significance of disturbed alpha oscillations. In line with this notion, studies investigating neurophysiological correlates of consciousness following administration of propofol or ketamine found similar global alpha band disconnectivity, which supports the notion of this finding as a correlate of disturbed consciousness [60, 61].

We also found regional changes of connectivity including enhanced theta band activity particularly in central and temporo-parietal regions. A general slowing of background activity is a well-known phenomenon in delirium, yet its pathophysiological relevance in delirium is unclear. Frontal midline theta oscillations were shown to be engaged in working memory, top-down cognitive control, and modulations of anxiety [62, 63]. Intrinsic theta activity was furthermore shown to constitute the functional architecture of top-down attention, which is consistent with impaired attention, increased impulsivity, and reduced verbal and visual memory when theta activity is pathologically enhanced [64–66]. Although increased theta activity is, as discussed above, an ambiguous finding in delirium studies, we clearly find connectivity in the theta band to be increased rendering it a tenable source of attentional and memory deficits. Given this preliminary evidence for delirium pathophysiology and medium to strong effect sizes, theta band hyperconnectivity seems to be similarly well-suited as alpha band disconnectivity as a biomarker of delirium.

Beta band connectivity was regionally disturbed, particularly in parieto-occipital regions. None of the previous studies investigated the significance of beta oscillations in delirium and the retrospective design of this study, which aimed to estimate effect sizes for planning prospective studies, rendered correlations with behavioral subtypes impossible. Most evidence in the literature yet points to a common role of beta band activity among cognitive processes which is the maintenance of an endogenous status quo [67]. This interpretation is highlighted by increased beta activity during the maintenance of steady-state force output or expectant immobility in the motor domain [68, 69]. In other domains, holding information required for an upcoming task, endogenous selection of relevant information from ambiguous stimuli, overriding distracting external stimuli, or making endogenously driven choices were also associated with an increase in beta power [70–73]. Decreased beta band connectivity may therefore be associated with impaired stability of cognitive processes, which is a well-known phenomenon among delirious patients. Effect sizes were only medium, which may indicate that only a subset of delirious patients, e.g., more severe delirium or due to a certain etiology, is affected by disturbed communication in the beta band.

### Source analyses and network findings

Betweenness centrality is a conceptually easy, common, and surprisingly robust parameter for characterizing the engagement of nodes within networks [74]. We found information flow to be altered in multiple nodes of all investigated networks. This discussion will focus on network-specific considerations beyond implications that relate to more global functions of oscillations, which were already discussed above. Effect sizes in network nodes were generally medium rendering them suitable for prospective evaluations of preliminary evidence from this study.

The default mode network is the most studied ICN and includes brain regions with dense functional connectivity such as the precuneus, medial prefrontal (MPFC), posterior cingulate, and parietal and mesial temporal cortices [75]. A fine-tuned homeostasis of slow, mostly theta, and fast oscillatory activity, especially in parietal and hippocampal regions, is considered relevant for working memory and memory consolidation [76]. Disturbances in this network are therefore a plausible correlate of working memory disturbances during and amnesia following an episode of delirium [77, 78]. Disturbed oscillatory activity in the MPFC, one of the key regions of the default mode network, is another plausible correlate for typical behavioral disturbances in delirium given its relevance for social behavior, mood control, and motivational drive [79]. A recent fMRI study including nine patients during an episode of delirium found a reduced betweenness in the right posterior cingulate cortex which may be interpreted as a deficient hub region that was also apparent in our study [28]. Van Montfort et al. furthermore found betweenness centrality to be reduced in the MPFC. In line with our findings of increased connectivity in the DMN, another fMRI study found increased functional connectivity between DLPFC and PCC during delirium compared to normal controls [24].

The salience network is regarded a system that integrates multisensory information with visceral and autonomic states to identify homeostatically relevant inputs and actions [80, 81]. In line with this notion, major parts of the network are constituted around paralimbic structures including the anterior cingulate cortex (ACC) and frontoinsular regions [82]. These regions were shown to be involved in interoceptive processing of anxiety, pain, and metabolic stress but also conflicts and errors [82–84]. It is somewhat unsurprising they also showed altered oscillatory activity in this study’s delirious population since delirium is associated with significant disturbances of endocrine and metabolic functions that are expected to affect interoceptive signaling [85]. Another important consequence of being delirious is the continuous presence of nocuous situations including pain, restraint, and thirst but also disorientation and uncertainty that would influence salience network structures [6]. In line with our findings in the salience network, a previous fMRI reported information flow to be disturbed in the ACC in delirium [28].

The executive control network constitutes, next to the salience network, the second ICN within the task-activation network [82]. It operates on identified salience and includes structures that serve sustained attention and maintenance of action relevant data in mind (lateral parietal cortex, DLPFC), control over sensorimotor representations (ventrolateral prefrontal cortex), and response selection (dorsomedial frontal cortex) [86–88]. Source analyses revealed altered betweenness centrality in multiple ECN nodes in the studied delirious population indicating impaired homeostasis in this network. Since it is increasingly understood that switching between distinct brain networks, such as the DMN and ECN, is an essential mechanism required for both exogenous and endogenous cognitive control, our findings pose a potential substrate of attentional deficits and incoherent action in delirium [89].

### Limitations

We cannot rule out a selection bias that is immanent to retrospective sample collection procedures. Although substantial efforts were made to include only EEG data from patients that had a confirmed diagnosis of delirium, it is possible that the fluctuating course of delirium severity may have caused routine EEGs not to be recorded when delirium was most severe. Another limitation is that the discussion of behavioral effects is not based on explicit data obtained from this study’s population but on typical features in delirious patients. We yet made substantial efforts to include only data of patients that were clearly diagnosed with an episode of delirium in line with current diagnostic criteria. Patients included in this study thus presented by definition with impaired attention, perception, and cognitive disturbances including memory, executive, and orientation domains.

### Implications for prospective studies

Delirium is the most common neuropsychiatric condition in hospitals. Yet it is a grossly underdiagnosed condition and there are no objective biomarkers to guide its clinical management [11, 90]. This study’s objective was to assess effect sizes of EEG measures of delirium and thereby substantiate future research investigating EEG biomarkers to improve diagnosis, treatment, and prognosis of affected patients. Our results clearly implicate that EEG is a promising method in this context given that multiple and specific biomarker candidates were identified. Effect sizes were medium to strong, which satisfies our definition of EEG measures being suitable for investigations in prospective trials and provides further support of the method. Strongest effects were found for theta and alpha band connectivity in networks engaged in working memory, sustained attention, and top-down control rendering these most suitable to be investigated in future studies. Given the evaluation of a mixed patient population, results are not confined to a specific cause of delirium and should be considered in all prospective evaluations of EEG biomarkers in delirium.

## Conclusions

This pilot study provides comprehensive evidence that EEG biomarkers are promising tools to advance research and enhance care in delirium. Altered homeostasis of oscillatory brain activity is a key finding and functional networks are critically disrupted in delirium, which may be central to clinical features. Source analyses revealed that multiple nodes of intrinsic connectivity networks tasked with cognitive functions such as working memory, salience detection, sustained attention, and executive control are affected during delirium. Effect sizes were generally medium to strong indicating that EEG-based connectivity and network analyses are viable means to elaborate on the pathophysiology of delirium. Results will be used for planning a prospective observational study investigating identified biomarker candidates regarding their therapeutic and prognostic significance.

## Additional files

Additional file 1: Search terms used for the retrospective identification of delirious patients. List of key words that were entered to the EEG documentation system in order to screen for delirious patients. (DOCX 12 kb)

Additional file 2: Flow chart of the patient selection procedure. Flow chart of the patient selection procedure in line with suggestions made by the STROBE guidelines for reporting of case-control studies. (DOCX 33 kb)

Additional file 3 Power differences between groups. Complete table of frequency band specific pairwise comparisons for power differences between groups. (PDF 21 kb)
