# Supplementary material for: Delirium is associated with frequency band specific dysconnectivity in intrinsic connectivity networks: preliminary evidence from a large retrospective pilot case-control study
Source: Pilot Feasibility Stud. 2019 Jan 7;5:2. doi: 10.1186/s40814-018-0388-z (PMC6322230; doi:10.1186/s40814-018-0388-z)
Supplement: Supplementary file 1 — Search terms used for the retrospective identification of delirious patients. List of key words that were entered to the EEG documentation system in order to screen for delirious patients. (DOCX 12 kb) [file 40814_2018_388_MOESM1_ESM.docx]

EEG sets contained information about indication, EEG description and results. Search terms for identification of patients with a diagnosis of delirium within the hospital EEG database are enlisted below. Resulting patients were carefully hand screened for eligibility for the DELIRIUM group.

Delir

verwirr*

bewusst*

bewußt*

Encephalopathie

Encephalitis

org. affektive Störung

org. Psychose

Wesensveränderung

Orientierungsstörung

kognitive Einbußen unklarer Ätiologie

Hirnfunktionsstörung

Desorientiertheit

Vigilanzminderung
