# Supplementary material for: Delirium is associated with frequency band specific dysconnectivity in intrinsic connectivity networks: preliminary evidence from a large retrospective pilot case-control study
Source: Pilot Feasibility Stud. 2019 Jan 7;5:2. doi: 10.1186/s40814-018-0388-z (PMC6322230; doi:10.1186/s40814-018-0388-z)
Supplement: Supplementary file 2 — Flow chart of the patient selection procedure. Flow chart of the patient selection procedure in line with suggestions made by the STROBE guidelines for reporting of case-control studies. (DOCX 33 kb) [file 40814_2018_388_MOESM2_ESM.docx]

**Database of EEG recordings** (years 2004-2016)

N= 9980

**Keyword Search** (e.g. “physiologic”, “normal”)

n = 2210

**Keyword search** (e.g.“delirium”, “conscious*,…) ^1,2^

n = 567

1

**Control Group**

N=414

**Delirium Group**

N=129

**Assessment of discharge letters**

Exclusion criteria: No clear diagnosis of delirium in accordance with DSM-5 criteria, imaging indicating structural brain lesions (stroke, tumor, inflammatory disease etc.)

**Assessment of EEG reports**

Exclusion criteria: EEGs limited by artifacts, medication or vigilance

n = 184

n = 1341

**Automated systematic age- and gender-Matching**

using custom MATLAB® scripts

Flowchart Patient Selection Process

^1^ asterisks indicate wildcard characters

^2^ for detailed search terms see Appendix A
