# Supplementary material for: Delirium is associated with frequency band specific dysconnectivity in intrinsic connectivity networks: preliminary evidence from a large retrospective pilot case-control study
Source: Pilot Feasibility Stud. 2019 Jan 7;5:2. doi: 10.1186/s40814-018-0388-z (PMC6322230; doi:10.1186/s40814-018-0388-z)
Supplement: Supplementary file 3 — Power differences between groups. Complete table of frequency band specific pairwise comparisons for power differences between groups. (PDF 21 kb) [file 40814_2018_388_MOESM3_ESM.pdf]

Pairwise Comparisons

Dependent Variable: Power

FREQBAND				Mean Difference (I-J)	Std. Error	Sig.[b]	95% Confidence Interval for Difference[b] Lower Bound	Upper Bound
Delta	Fp2	Control	Delirium	-32,001*	,632	0,000	-33,240	-30,762
		Delirium	Control	32,001*	,632	0,000	30,762	33,240
	Fp1	Control	Delirium	-42,394*	,632	0,000	-43,633	-41,154
		Delirium	Control	42,394*	,632	0,000	41,154	43,633
	F8	Control	Delirium	-25,718*	,632	0,000	-26,958	-24,479
		Delirium	Control	25,718*	,632	0,000	24,479	26,958
	F4	Control	Delirium	-4,060*	,632	,000	-5,299	-2,821
		Delirium	Control	4,060*	,632	,000	2,821	5,299
	Fz	Control	Delirium	-6,259*	,632	,000	-7,498	-5,020
		Delirium	Control	6,259*	,632	,000	5,020	7,498
	F3	Control	Delirium	-8,380*	,632	,000	-9,619	-7,141
		Delirium	Control	8,380*	,632	,000	7,141	9,619
	F7	Control	Delirium	-22,900*	,632	,000	-24,139	-21,661
		Delirium	Control	22,900*	,632	,000	21,661	24,139
	T4	Control	Delirium	-6,226*	,632	,000	-7,465	-4,987
		Delirium	Control	6,226*	,632	,000	4,987	7,465
	C4	Control	Delirium	,027	,632	,965	-1,212	1,266
		Delirium	Control	-,027	,632	,965	-1,266	1,212
	Cz	Control	Delirium	-1,009	,632	,110	-2,248	,230
		Delirium	Control	1,009	,632	,110	-,230	2,248
	C3	Control	Delirium	,294	,632	,642	-,945	1,533
		Delirium	Control	-,294	,632	,642	-1,533	,945
	T3	Control	Delirium	-2,407*	,632	,000	-3,646	-1,168
		Delirium	Control	2,407*	,632	,000	1,168	3,646
	T6	Control	Delirium	-7,561*	,632	,000	-8,800	-6,322
		Delirium	Control	7,561*	,632	,000	6,322	8,800
	P4	Control	Delirium	,326	,632	,606	-,913	1,565
		Delirium	Control	-,326	,632	,606	-1,565	,913
	Pz	Control	Delirium	-,899	,632	,155	-2,138	,340
		Delirium	Control	,899	,632	,155	-,340	2,138
	P3	Control	Delirium	2,058*	,632	,001	,818	3,297
		Delirium	Control	-2,058*	,632	,001	-3,297	-,818
	T5	Control	Delirium	-2,860*	,632	,000	-4,099	-1,621
		Delirium	Control	2,860*	,632	,000	1,621	4,099
	O2	Control	Delirium	-4,974*	,632	,000	-6,213	-3,734
		Delirium	Control	4,974*	,632	,000	3,734	6,213
	O1	Control	Delirium	-4,015*	,632	,000	-5,254	-2,776
		Delirium	Control	4,015*	,632	,000	2,776	5,254
Theta	Fp2	Control	Delirium	-9,003*	,632	,000	-10,242	-7,764
		Delirium	Control	9,003*	,632	,000	7,764	10,242
	Fp1	Control	Delirium	-14,456*	,632	,000	-15,695	-13,217
		Delirium	Control	14,456*	,632	,000	13,217	15,695
	F8	Control	Delirium	-9,244*	,632	,000	-10,483	-8,005
		Delirium	Control	9,244*	,632	,000	8,005	10,483
	F4	Control	Delirium	-,256	,632	,686	-1,495	,983
		Delirium	Control	,256	,632	,686	-,983	1,495
	Fz	Control	Delirium	-1,954*	,632	,002	-3,193	-,715
		Delirium	Control	1,954*	,632	,002	,715	3,193
	F3	Control	Delirium	-2,942*	,632	,000	-4,181	-1,703
		Delirium	Control	2,942*	,632	,000	1,703	4,181
	F7	Control	Delirium	-8,086*	,632	,000	-9,325	-6,847

		Delirium	Control	8,086*	,632	,000	6,847	9,325
	T4	Control	Delirium	-2,133*	,632	,001	-3,372	-,894
		Delirium	Control	2,133*	,632	,001	,894	3,372
	C4	Control	Delirium	,589	,632	,351	-,650	1,828
		Delirium	Control	-,589	,632	,351	-1,828	,650
	Cz	Control	Delirium	,617	,632	,329	-,622	1,856
		Delirium	Control	-,617	,632	,329	-1,856	,622
	C3	Control	Delirium	1,128	,632	,074	-,111	2,367
		Delirium	Control	-1,128	,632	,074	-2,367	,111
	T3	Control	Delirium	,057	,632	,928	-1,182	1,297
		Delirium	Control	-,057	,632	,928	-1,297	1,182
	T6	Control	Delirium	-1,654*	,632	,009	-2,893	-,415
		Delirium	Control	1,654*	,632	,009	,415	2,893
	P4	Control	Delirium	1,111	,632	,079	-,129	2,350
		Delirium	Control	-1,111	,632	,079	-2,350	,129
	Pz	Control	Delirium	2,986*	,632	,000	1,747	4,225
		Delirium	Control	-2,986*	,632	,000	-4,225	-1,747
	P3	Control	Delirium	3,424*	,632	,000	2,185	4,663
		Delirium	Control	-3,424*	,632	,000	-4,663	-2,185
	T5	Control	Delirium	1,439*	,632	,023	,200	2,678
		Delirium	Control	-1,439*	,632	,023	-2,678	-,200
	O2	Control	Delirium	,639	,632	,312	-,600	1,878
		Delirium	Control	-,639	,632	,312	-1,878	,600
	O1	Control	Delirium	1,033	,632	,102	-,206	2,272
		Delirium	Control	-1,033	,632	,102	-2,272	,206
Alpha	Fp2	Control	Delirium	1,222	,632	,053	-,017	2,461
		Delirium	Control	-1,222	,632	,053	-2,461	,017
	Fp1	Control	Delirium	-,825	,632	,192	-2,064	,414
		Delirium	Control	,825	,632	,192	-,414	2,064
	F8	Control	Delirium	-,859	,632	,174	-2,098	,380
		Delirium	Control	,859	,632	,174	-,380	2,098
	F4	Control	Delirium	1,399*	,632	,027	,160	2,638
		Delirium	Control	-1,399*	,632	,027	-2,638	-,160
	Fz	Control	Delirium	,320	,632	,613	-,919	1,559
		Delirium	Control	-,320	,632	,613	-1,559	,919
	F3	Control	Delirium	-,052	,632	,935	-1,291	1,187
		Delirium	Control	,052	,632	,935	-1,187	1,291
	F7	Control	Delirium	-,539	,632	,394	-1,778	,700
		Delirium	Control	,539	,632	,394	-,700	1,778
	T4	Control	Delirium	,008	,632	,990	-1,231	1,247
		Delirium	Control	-,008	,632	,990	-1,247	1,231
	C4	Control	Delirium	,662	,632	,295	-,577	1,901
		Delirium	Control	-,662	,632	,295	-1,901	,577
	Cz	Control	Delirium	1,422*	,632	,025	,182	2,661
		Delirium	Control	-1,422*	,632	,025	-2,661	-,182
	C3	Control	Delirium	1,631*	,632	,010	,391	2,870
		Delirium	Control	-1,631*	,632	,010	-2,870	-,391
	T3	Control	Delirium	1,315*	,632	,038	,076	2,554
		Delirium	Control	-1,315*	,632	,038	-2,554	-,076
	T6	Control	Delirium	1,268*	,632	,045	,029	2,507
		Delirium	Control	-1,268*	,632	,045	-2,507	-,029
	P4	Control	Delirium	1,471*	,632	,020	,232	2,710
		Delirium	Control	-1,471*	,632	,020	-2,710	-,232
	Pz	Control	Delirium	5,082*	,632	,000	3,843	6,321
		Delirium	Control	-5,082*	,632	,000	-6,321	-3,843
	P3	Control	Delirium	4,925*	,632	,000	3,686	6,164
		Delirium	Control	-4,925*	,632	,000	-6,164	-3,686

	T5	Control	Delirium	4,621*	,632	,000	3,382	5,860
		Delirium	Control	-4,621*	,632	,000	-5,860	-3,382
	O2	Control	Delirium	4,389*	,632	,000	3,150	5,628
		Delirium	Control	-4,389*	,632	,000	-5,628	-3,150
	O1	Control	Delirium	4,086*	,632	,000	2,847	5,325
		Delirium	Control	-4,086*	,632	,000	-5,325	-2,847
Beta	Fp2	Control	Delirium	,313	,632	,621	-,926	1,552
		Delirium	Control	-,313	,632	,621	-1,552	,926
	Fp1	Control	Delirium	-,029	,632	,963	-1,268	1,210
		Delirium	Control	,029	,632	,963	-1,210	1,268
	F8	Control	Delirium	,003	,632	,997	-1,236	1,242
		Delirium	Control	-,003	,632	,997	-1,242	1,236
	F4	Control	Delirium	,422	,632	,504	-,817	1,661
		Delirium	Control	-,422	,632	,504	-1,661	,817
	Fz	Control	Delirium	,194	,632	,759	-1,045	1,433
		Delirium	Control	-,194	,632	,759	-1,433	1,045
	F3	Control	Delirium	,173	,632	,785	-1,066	1,412
		Delirium	Control	-,173	,632	,785	-1,412	1,066
	F7	Control	Delirium	,047	,632	,941	-1,192	1,286
		Delirium	Control	-,047	,632	,941	-1,286	1,192
	T4	Control	Delirium	,158	,632	,803	-1,081	1,397
		Delirium	Control	-,158	,632	,803	-1,397	1,081
	C4	Control	Delirium	,239	,632	,706	-1,000	1,478
		Delirium	Control	-,239	,632	,706	-1,478	1,000
	Cz	Control	Delirium	,435	,632	,491	-,804	1,674
		Delirium	Control	-,435	,632	,491	-1,674	,804
	C3	Control	Delirium	,507	,632	,422	-,732	1,747
		Delirium	Control	-,507	,632	,422	-1,747	,732
	T3	Control	Delirium	,465	,632	,462	-,774	1,704
		Delirium	Control	-,465	,632	,462	-1,704	,774
	T6	Control	Delirium	,452	,632	,474	-,787	1,691
		Delirium	Control	-,452	,632	,474	-1,691	,787
	P4	Control	Delirium	,461	,632	,466	-,778	1,700
		Delirium	Control	-,461	,632	,466	-1,700	,778
	Pz	Control	Delirium	,815	,632	,198	-,424	2,054
		Delirium	Control	-,815	,632	,198	-2,054	,424
	P3	Control	Delirium	,908	,632	,151	-,331	2,147
		Delirium	Control	-,908	,632	,151	-2,147	,331
	T5	Control	Delirium	,849	,632	,179	-,390	2,088
		Delirium	Control	-,849	,632	,179	-2,088	,390
	O2	Control	Delirium	,854	,632	,177	-,385	2,093
		Delirium	Control	-,854	,632	,177	-2,093	,385
	O1	Control	Delirium	,720	,632	,255	-,520	1,959
		Delirium	Control	-,720	,632	,255	-1,959	,520
Gamma	Fp2	Control	Delirium	,081	,632	,898	-1,158	1,320
		Delirium	Control	-,081	,632	,898	-1,320	1,158
	Fp1	Control	Delirium	,020	,632	,975	-1,219	1,259
		Delirium	Control	-,020	,632	,975	-1,259	1,219
	F8	Control	Delirium	,082	,632	,897	-1,157	1,321
		Delirium	Control	-,082	,632	,897	-1,321	1,157
	F4	Control	Delirium	,119	,632	,850	-1,120	1,358
		Delirium	Control	-,119	,632	,850	-1,358	1,120
	Fz	Control	Delirium	,090	,632	,887	-1,149	1,329
		Delirium	Control	-,090	,632	,887	-1,329	1,149
	F3	Control	Delirium	,069	,632	,913	-1,170	1,308
		Delirium	Control	-,069	,632	,913	-1,308	1,170
	F7	Control	Delirium	,010	,632	,988	-1,229	1,249

		Delirium	Control	-,010	,632	,988	-1,249	1,229
	T4	Control	Delirium	,070	,632	,911	-1,169	1,310
		Delirium	Control	-,070	,632	,911	-1,310	1,169
	C4	Control	Delirium	,121	,632	,848	-1,118	1,360
		Delirium	Control	-,121	,632	,848	-1,360	1,118
	Cz	Control	Delirium	,105	,632	,868	-1,134	1,344
		Delirium	Control	-,105	,632	,868	-1,344	1,134
	C3	Control	Delirium	,079	,632	,901	-1,160	1,318
		Delirium	Control	-,079	,632	,901	-1,318	1,160
	T3	Control	Delirium	,067	,632	,916	-1,172	1,306
		Delirium	Control	-,067	,632	,916	-1,306	1,172
	T6	Control	Delirium	,042	,632	,947	-1,197	1,281
		Delirium	Control	-,042	,632	,947	-1,281	1,197
	P4	Control	Delirium	,126	,632	,842	-1,113	1,365
		Delirium	Control	-,126	,632	,842	-1,365	1,113
	Pz	Control	Delirium	,132	,632	,835	-1,108	1,371
		Delirium	Control	-,132	,632	,835	-1,371	1,108
	P3	Control	Delirium	,089	,632	,888	-1,150	1,328
		Delirium	Control	-,089	,632	,888	-1,328	1,150
	T5	Control	Delirium	,019	,632	,976	-1,220	1,258
		Delirium	Control	-,019	,632	,976	-1,258	1,220
	O2	Control	Delirium	,025	,632	,969	-1,214	1,264
		Delirium	Control	-,025	,632	,969	-1,264	1,214
	O1	Control	Delirium	,083	,632	,896	-1,156	1,322
		Delirium	Control	-,083	,632	,896	-1,322	1,156

Based on estimated marginal means

*. The mean difference is significant at the 0,05 level.

b. Adjustment for multiple comparisons: Bonferroni.
